# The Normal and Brain Tumor Vasculature: Morphological and Functional Characteristics and Therapeutic Targeting

**DOI:** 10.3389/fphys.2021.622615

**Published:** 2021-03-05

**Authors:** Joris Guyon, Candice Chapouly, Laetitia Andrique, Andreas Bikfalvi, Thomas Daubon

**Affiliations:** ^1^INSERM, LAMC, U1029, University Bordeaux, Pessac, France; ^2^INSERM, Biology of Cardiovascular Diseases, U1034, University Bordeaux, Pessac, France; ^3^VoxCell 3D Plateform, UMS TBMcore 3427, Bordeaux, France; ^4^University Bordeaux, CNRS, IBGC, UMR 5095, Bordeaux, France

**Keywords:** brain, glioblastoma, blood vessels, astrocyte, antiangiogenic therapy, vascular tissue engineering

## Abstract

Glioblastoma is among the most common tumor of the central nervous system in adults. Overall survival has not significantly improved over the last decade, even with optimizing standard therapeutic care including extent of resection and radio- and chemotherapy. In this article, we review features of the brain vasculature found in healthy cerebral tissue and in glioblastoma. Brain vessels are of various sizes and composed of several vascular cell types. Non-vascular cells such as astrocytes or microglia also interact with the vasculature and play important roles. We also discuss *in vitro* engineered artificial blood vessels which may represent useful models for better understanding the tumor–vessel interaction. Finally, we summarize results from clinical trials with anti-angiogenic therapy alone or in combination, and discuss the value of these approaches for targeting glioblastoma.

## Introduction

In vertebrates, vessels are built of an internal layer made of endothelial cells which are in contact with the blood and of mural cells that are composed of either smooth muscle cells (larger vessels) or pericytes (in capillaries). In the brain, blood vessels are tightly organized and participate in blood and brain tissue exchange via the blood–brain barrier (BBB).

Within the brain tumor vasculature, two different types of vessels are found, vessels formed by angiogenesis (neoangiogenic vessels) and preexisting vessels which may be co-opted by tumor cells (co-opted vessels). Anti-angiogenic strategies have been developed for targeting the brain tumor vasculature (Lakka and Rao, 2008). Unfortunately, clinical trials were not crowned with success (Chinot et al., 2014). A contributing factor is represented by a shift of tumor cells to a co-optive mode induced by anti-angiogenic therapy which contributes to tumor spread and development (Griveau et al., 2018).

In this article, we discuss the characteristics and specific features of the normal and brain tumor vasculature. We will also include in our discussion *in vitro* constructed artificial blood vessels by tissue engineering which represent an interesting tool to study tissue–vessel interactions and may also be useful in the tumor context. Finally, we will review some recent clinical studies using anti-angiogenic drugs in glioblastoma.

## The Blood–Brain Barrier

**FIGURE 1:**
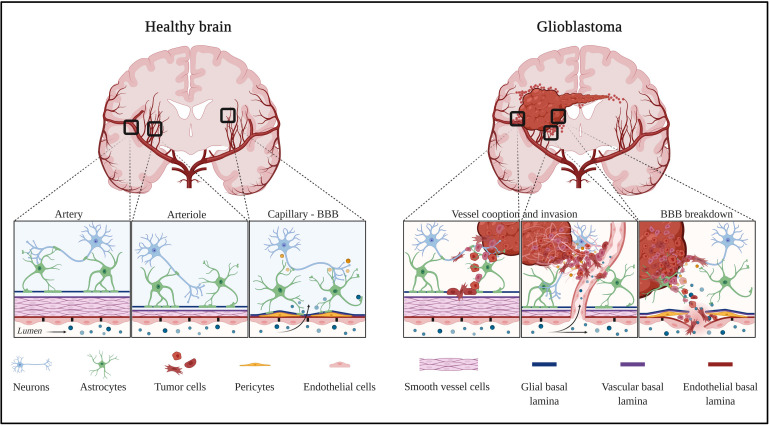
Healthy and tumor brain vascular architecture: focus on artery, arteriole, and capillary. **Left panel:** in a healthy brain vasculature, endothelial cell monolayer is surrounded by a smooth muscle coat in arteries and arterioles, and is replaced by pericytes in the capillaries. The perivascular space is delimited by the vascular basement membrane and glial basement membrane. This space gradually diminished and the two basement membranes enter in direct contact to astrocytes endfeet. Molecules diffuse or are transported at the capillary level. **Right panel:** GBM is a highly angiogenic and infiltrative tumor. Cells invade along blood vessels to support tumor growth (co-option). GBM displaces astrocytes endfeet and alters pericyte stability, leading to perivascular niches and cell evasion. Created with Biorender.com.

In a healthy individual, the central nervous system (CNS) parenchyma is protected from the peripheral circulation by the BBB. This barrier comprises a network of blood vessels made of endothelial cells with unique features (Figure 1 – healthy brain). Endothelial cells at the BBB act as gatekeepers to control soluble factors and immune cell trafficking into the vessel wall and underlying tissues, and both the transcellular and paracellular pathways are involved in this process.

### The Paracellular Pathway

The paracellular pathway is modulated by the coordinated opening and closure of endothelial cell–cell junctions which involves a complex rearrangement of endothelium-specific transmembrane tight/adherens junction proteins and the related cytoskeleton. CNS tight junctions are primarily formed by Claudin5 and Occludin, and are coupled with the Zonula Occludens intracellular proteins (ZO1, ZO2, and ZO3) which form a scaffold between these transmembrane proteins and the actin cytoskeleton. Other Claudins may be involved in controlling endothelial cell paracellular barrier properties, as Claudin5 downregulation only leads to small size molecule leakage at the BBB (Nitta et al., 2003). Claudin1, Claudin3, and Claudin12 have been identified as potential candidates, but their role still needs to be clarified (Ohtsuki et al., 2008; Daneman et al., 2010a; Kooij et al., 2014). Members of the immunoglobulin superfamily, notably CAMs (PECAM1, ICAM1, and VCAM1), JAMs (JAM1–3), and Nectin proteins, clustered at CNS endothelial intercellular contacts, promote homotypic cell–cell adhesion and regulate inflammatory cell transmigration at the BBB (Del Maschio et al., 1999). Recent literature suggested that tricellular contacts, where the corners of three cells meet, can also be found in CNS endothelial cells. Specifically, tricellulin and angulin-1/lipolysis-stimulated lipoprotein receptor (LSR) were shown to participate to BBB paracellular control of plasmatic protein and immune cell trafficking (Iwamoto et al., 2014; Sohet et al., 2015). Importantly, tight junctions cannot be dissociated from adherent junctions in regulating BBB tightness; CNS endothelial adherent junctions are characterized by homophilic cadherin (VE-cadherin and *N*-cadherin) interactions controlling cell adhesion. Adherent junctions are linked to the cytoskeleton *via* their binding partner β-catenin and participate to BBB tightness through phosphorylation, cleavage, and internalization, or by modulating Claudin5 expression level (Meng and Takeichi, 2009).

### The Transcellular Pathway

Aside from the paracellular pathway, brain nutrient intake and CNS toxin removal are highly regulated by solute transporters and receptor-mediated transcytosis at the BBB, and inflammatory cells are actively prevented from crossing the BBB by low levels of immune receptors that normally permit immune trafficking.

Specifically, the cerebral endothelium expresses a number of specific solute transporters to facilitate the carrier-mediated transport of glucose (glucose transporters: GLUT1), amino acids (cationic amino acid transporters: CAT1, CAT3), monocarboxylic acids (monocarboxylate transporters: MCT1), hormones (thyroid hormone transporters: MCT8), fatty acids (fatty acid transporters: FATP-1), nucleotides (nucleoside transporters: ENT1, ENT2), ions (organic anion and organic cation transporters), amines, choline, and vitamins, which are otherwise excluded from the brain due to the paracellular pathway (Sweeney et al., 2019). Efflux mechanisms also contribute to barrier functions, with ATP-binding cassette transporters (P-gp: MDR1), breast cancer resistance protein (BCRP), and multidrug resistance-associated proteins (MRP 1–5) hydrolyzing ATP to actively pump drugs and their conjugates, xenobiotics, endogenous metabolites, and nucleosides across the luminal side of blood vessels into the circulation (Mahringer and Fricker, 2016; Sweeney et al., 2019).

Selective peptides and even large proteins can enter the brain by binding to receptors on endothelial cells via endocytosis (receptor-mediated transcytosis) (leptin receptors, transferrin receptors, and insulin receptors) (Preston et al., 2014). The BBB is characterized by extremely low rates of vesicular transport (transcytosis) which limits the transcellular passage across CNS barriers. It has been recently reported that the major facilitator superfamily domain containing 2a (Mfsd2a) is selectively expressed in CNS blood vessels and actively participates to BBB homeostasis by suppressing transcytosis in CNS endothelial cells (Ben-Zvi et al., 2014). Mfsd2a is located upstream of Caveolin-1 (Cav-1) (Chow and Gu, 2017), which is involved in regulating endothelial permeability, angiogenesis, and leukocyte diapedesis (Zhao et al., 2014), and VE-cadherin/catenin complex targets Cav-1 to endothelial cell junctions leading to BBB breakdown under permeability conditions (Kronstein et al., 2012).

These features exist within the majority of the CNS capillary population. However, it is worth noting that some regions of the CNS display a leaky BBB. These regions are grouped together under the term “circumventricular organs” which are regions of the brain sensing blood-borne signals. Morphologically, in the circumventricular organs, the capillaries are fenestrated with discontinuous tight junctions and thinner endothelial cells which contain more vesicles than capillaries of other parts of the CNS (Coomber and Stewart, 1985). This permits the two-way exchange of metabolic information: the delivery of neuro-hormones into the bloodstream by secretory organs and the sensing of blood-borne molecules by neurons in sensory organs. However, within the circumventricular organs, there is no direct passage of blood-borne substances in the parenchyma due to the presence of outer basement membrane but also astrocytes and tanycytes (ependymal cells sharing common features with radial glial cells and astrocytes) which are considered alternative CNS barriers (Langlet et al., 2013). The peculiar organization of the BBB within the circumventricular organs leads us to approach the CNS vascular barrier system from another angle by acknowledging its complex architecture which is not just a vascular endothelium. Indeed, it is more proper to consider it as a multi-cellular neurovascular unit comprising notably astrocytes, pericytes, basement membranes as well as blood vessels.

## The Neurovascular Unit, a Multi-Cellular CNS-Barrier Structure

A wealth of literature published during the last decades has enabled a change in the vision of the BBB, leading to the concept of a multi-cellular CNS-barrier structure. Indeed, substantial intercellular communication occurs between vascular cells (endothelial cells and pericytes) and the adjoining glia (Iadecola, 2017). More specifically, to enter the CNS from the vasculature, soluble factors and immune cells must traverse the endothelial BBB and the adjacent pericyte layer. Once soluble factors and immune cells penetrate the BBB, they circulate within the perivascular space, a region surrounding the basal surface of the endothelial cell wall, to reach the CNS parenchyma by passing through the glia limitans composed of the parenchymal basement membrane and the astrocyte endfeet (Engelhardt and Coisne, 2011; Engelhardt and Ransohoff, 2012).

### Pericytes

Pericytes, strategically positioned along capillaries, play a critical role in the multi-cellular CNS barrier structure. Indeed, pericytes, sandwiched between endothelial cells and astrocytes, are dynamically and synergistically engaged in interactions with neighboring cells to maintain homeostasis of the CNS (Figure 1 – healthy brain). Pericytes are notably involved in the regulation of cerebral blood flow, neurovascular coupling and BBB homeostasis.

A role for pericytes in the regulation of microcirculatory blood flow has long been suspected (Armulik et al., 2011; Hall et al., 2014; Sweeney et al., 2016). Recent work demonstrated that pericytes synchronize microvascular blood flow dynamics and neurovascular coupling *via* nanotube-like processes called inter-pericyte tunneling nanotubes (IP-TNTs) which connect two pericytes on separate capillaries to form a functional network (Alarcon-Martinez et al., 2020). Pericytes are also part of the neurovascular unit and are most firmly attached to brain capillaries. They are involved in a crosstalk between endothelial cells and the surrounding cerebral tissue. Notably, it has been reported that pericytes interact with endothelial cells via specific adhesion sites that represent peg-and-socket junctions in the presence of *N*-cadherin (Sweeney et al., 2016), the single cell adhesion receptor CD146 (Chen et al., 2017), adhesion plaques containing fibronectin (Courtoy and Boyles, 1983), Connexin43 gap junctions (Cuevas et al., 1984), and even tight junctions (Larson et al., 1987). In a mouse model lacking pericyte coverage at the microvascular level, BBB integrity is compromised because of the transcellular barrier specific dysfunction (Sweeney et al., 2016) but also because of the loss of astrocytic endfeet polarization (Armulik et al., 2010). While pericytes are necessary for maintaining BBB integrity, astrocytic endfeets are also major actors in CNS barrier homeostasis.

### Astrocytes

Astrocytes represent an important population of glial cells in the CNS and astrocytic endfeet create a thick continuous layer that covers BBB microvessels called the glia limitans (Mathiisen et al., 2010) (Figure 1 – healthy brain). Neural precursor cells represent the primary source of astrocytes which develop at late gestation stages in mammals (Daneman et al., 2010b; Cheslow and Alvarez, 2016). Therefore, it is commonly accepted that if astrocytes do not play a major role in BBB establishment, they strongly impact BBB maturation and maintenance.

Reducing the vascular coverage of the population of astrocytes and astrocytic endfeet in the early postnatal cerebral cortex leads to the formation of microvessels with an abnormally large diameter (Ma et al., 2012). In addition, astrocytes are actively involved in the production of the basement membrane embedding the glia limitans. Knocking down elements of the basement membrane results in the disruption of Aquaporin4 (AQP4) channel enrichment at the astrocytic endfeet membrane (Brightman, 2002; Lien et al., 2012; Menezes et al., 2014). This leads to BBB permeability and associated brain edema (Nagelhus and Ottersen, 2013).

Astrocytes maintain BBB integrity *via* the secretion of soluble factors. Astrocytes improve endothelial barrier function in co-culture or after administration of conditioned medium to CNS endothelial mono-culture (Igarashi et al., 1999; Alvarez et al., 2011; Podjaski et al., 2015). Astrocytes secrete soluble factors notably Sonic Hedgehog (Shh) (Alvarez et al., 2011), retinoic acid (RA) (Mizee et al., 2014), glial-derived neurotrophic factor (GDNF) (Igarashi et al., 1999), and angiopoietin 1 (Ang-1) (Suri et al., 1996; Pfaff et al., 2006) which reduce permeability.

Blood–brain barrier integrity in the cerebellum, spinal cord, and olfactory bulbs relies on astrocyte-derived Wnt-like ligand Norrin which interacts with the endothelial Frizzled4 receptor. Knocking down the Norrin/Frizzled4 signaling leads to BBB defects through β-catenin–dependent transcriptional regulation (Zhou et al., 2014).

Astrocytes also express members of the ephrin receptor (EphR)/ephrin family (Nestor et al., 2007) which may impact BBB homeostasis. Notably, EphA4 receptor is expressed by glial cells especially around blood vessels in the adult marmoset (Goldshmit et al., 2014). EphA4 also plays a role in vascular formation and guidance during CNS development in mice. The proper interaction between the EphA4 receptor and its astrocytic ephrinA5 ligand is necessary for the development of a normal vascular system in the hippocampus of adult mice (Goldshmit et al., 2006; Hara et al., 2010).

Hence, based on these strong arguments, it is now recognized that the vascular component in the CNS is inseparably linked to glial and neuronal partners. Therefore, it is necessary to consider the neurovascular unit as a whole (and not only the BBB) in vascular pathophysiology and targeted therapeutic strategies.

### Glioblastoma Disrupts The Normal Brain Architecture And Molecular Interactions

#### Basic Characteristics of Glioblastoma

Diffuse gliomas are brain tumors classified into IDH1mut and WT tumors (Louis et al., 2016). Glioblastomas (grade IV gliomas) are generally IDH WT tumors and represent the most aggressive form with an extremely poor prognosis. It is now admitted that glioblastoma (GBM) are mainly derived from neural stem cells, giving rise to transformed cells with astrocytic, neuronal, or oligodendrocytic characteristics (Alcantara Llaguno et al., 2009; Zong et al., 2012). Accumulation of genetic mutations, alterations, and amplifications play a central role in the transformation of healthy neural stem cells. Typical alterations in primary GBM are represented by amplification or mutation of PTEN, NF1, CDKN2A/B, and RB genes, or homozygous deletion or mutation of MDM2, CDK4, EGFR, PDGFRα, and PI3K genes. Typical histopathological features of GBM comprise highly proliferative cells with multinuclei, areas of necrosis surrounded by pseudopalisading cells, and endothelial cell proliferation with numerous clusters of blood vessels forming so-called glomeruloid structures. In 1938, Scherer highlighted several modes of GBM invasion: interstitial invasion, white matter tract invasion, and perivascular invasion (Scherer, 1940). GBM invasion relies on genetic alterations such as overexpression, amplification, deletion, or mutation in focal adhesion kinase (FAK) and phosphatidylinositol 3-kinase (PI3K) pathways. Activation of growth factors and their receptors are mainly involved in promoting invasion. These include CD44, integrins, osteonectin (SPARC), transforming growth factor (TGF)α/β, and receptors for platelet-derived growth factor (PDGF), fibroblast growth factor (FGF-2), and epidermal growth factor (EGF). Extracellular matrix components such as thrombospondins, laminins, or fibronectin are also overexpressed in GBM and their inhibition reduces invasiveness of GBM cells (Serres et al., 2014; Chouleur et al., 2020). Indeed, we and others characterized the role of thrombospondin-1 (which was primarily described as anti-angiogenic molecule) in GBM development and invasion (Daubon et al., 2019).

Glioblastoma is the most common brain tumor in Europe, in the United States, or in China, with more than 50% of glioma cases each year, and with an incidence of 3.2 per 100,000 people each year in the United States. Increasing incidence in populations from several countries were observed, suggested by authors as consequences of environmental or lifestyle factors (Philips et al., 2018). The 5-year overall survival (OS) rate is very low of only 5.1%, even with standard-of-care treatment (large tumor resection, chemo- and radiotherapy, so-called Stupp protocol). GBM are often only diagnosed at an advanced stage of the disease and often only detected when patients present symptoms (headaches, seizures, memory loss, loss of movements, cognitive impairments, and language dysfunctions). The poor response to therapy is partially explained by high intratumor heterogeneity, leaky and tortuous blood vessels in the central part, and intact BBB surrounding invasive cells, which leads to difficulties for therapeutic molecules to reach these sites (Figure 1 – glioblastoma).

### Metabolic Interactions Between GBM Cells and the Endothelium

Glioblastoma is considered as one of the most glycolytic human tumors. High glycolytic flux drive production of pyruvate from glucose, and then pyruvate into lactate by lactate dehydrogenases, to regenerate NAD^+^ to support glycolytic flux by fulfilling the demand for ATP and other metabolic precursors. As previously described in striated muscles and also in the brain, lactate is, in turn, retro-converted into pyruvate by oxygenated tumor cells to feed oxidative metabolism. This phenomenon was described in the seminal publication of Pellerin et al. (1998) as lactate shuttle between astrocytes and neurons. Vegran et al. (2011) also demonstrated a lactate shuttle between tumor cells and endothelial cells, mainly *via* endothelial monocarboxylate transporter 1 (MCT1). More recently, MCT1 was also identified as a key mediator of lactate signaling between glioma cells and brain endothelial cells (Miranda-Gonçalves et al., 2017). Targeting symbiotic metabolism between GBM and endothelial cells may represent an interesting therapeutic strategy.

### Involvement of Pericytes and Astrocytes in Glioblastoma Vascular Pathophysiology

The chronic hyper-permeability of blood vessels is a hallmark of glioblastoma. We focus herein our attention on the role of pericytes and astrocytes in disrupting the BBB in glioblastoma.

#### Role of Pericytes

Glioblastoma vessels are characterized by numerous structural and functional abnormalities, including altered association between endothelial cells and pericytes. These dysfunctional, unstable vessels contribute to hypoxia, interstitial fluid pressure, and enhanced susceptibility to metastatic invasion (Barlow et al., 2013). An interesting feature of glioblastoma pericytes is that they represent one of the active cell components of the perivascular niche. It has been reported that cancer stem cells, which are closely associated with tumor vessels, trans-differentiate into endothelial cells or pericytes (Wang et al., 2010; Cheng et al., 2013), a phenomenon described as vasculogenic mimicry (VM). VM was also shown to be promoted by tumor-associated macrophages (TAMs) by increasing the expression of cyclooxygenase 2 in the tumor cells (Rong et al., 2016) and has been associated with poor patient prognosis. However, the significance of VM in GBM is still debated and not universally accepted. Furthermore, tumor-derived pericytes exhibit specific genetic alterations that allow for discrimination between them and normal pericytes (Cheng et al., 2013), which may be relevant for diagnosis and therapy. Finally, pericytes were shown to promote evasion from the anti-tumor immune response favoring tumor growth (Figure 1 – glioblastoma). Glioblastoma-dependent immunosuppressive function in pericytes is mediated by the expression of anti-inflammatory molecules such as IL-10, TGF-β, and MHC-II (Valdor et al., 2017).

#### Role of Astrocytes

Reactive astrocytes are an integral part of the glioblastoma micro-environment and are characterized by hypertrophy, upregulation of intermediate filaments (vimentin and glial fibrillary acidic protein), and increase in proliferation. The role of reactive astrocytes in the pathophysiology of glioblastoma has been widely documented in the literature. Astrocyte–glioma crosstalk was shown to drive migration, proliferation, and invasion of glioblastoma (Guan et al., 2018). However, only few works focused on the contribution of astrocytes to the aberrant organization of the BBB in these tumors.

The participation of astrocytes to BBB permeability in glioblastoma is documented by the loss of astrocytic endfeet polarity which is characterized by Aquaporin-4 (AQP4) redistribution to membrane domains apart from endfeet areas (Kröger et al., 2004). This re-localization is probably due to the degradation of the proteoglycan agrin by the matrix metalloproteinase 3 (MMP3). Consequently, the water transport is compromised leading to edema. This, in turn, may drive BBB breakdown characterized by disrupted tight junctions leading to the development of vasogenic edema. However, how the loss of polarity is linked to the disturbance of microvascular tight junctions is still not understood (Wolburg et al., 2012). Using a clinically relevant mouse model of glioblastoma, it has been shown that tumor cells populate the perivascular space of preexisting vessels and displace astrocytic endfeet from endothelial or mural cells. This leads to abnormal BBB permeability and loss of astrocyte-mediated gliovascular coupling which pave the way for glioma cells to take control of vascular tone regulation (Watkins et al., 2014). This phenomenon, known as blood vessel co-option, is a strategy for glioblastoma to invade distant sites of the brain parenchyma (Figure 1 – glioblastoma). Vessel co-opting GBM cells directly obtain oxygen and nutrients from the blood. The interactions with the vascular niche stimulate proliferation and self-renewal of GBM cells.

There is still much to explore as reactive astrocytes have already been identified as key players impacting the state of the BBB in various diseases (Liebner et al., 2018). They are likely to also play an important role in vessel hyper-permeability of GBM.

## Vascular Tissue Engineering and Its Potential for the Study of GBM–Vessel Interactions

The tumor vasculature is critically involved in GBM development. This has led to clinical trials using anti-angiogenic drugs but with mixed results (Chinot et al., 2014). It has been postulated that anti-angiogenic treatment may impact tumor cell behavior by shifting them from an angiogenic to a co-optive behavior (Griveau et al., 2018). Thus, it is important to better understand more rigorously how tumor cells and vessels interact. A number of experimental models have been proposed in this context.

There are two classic and widely used models to study the role of the vasculature in tumor growth: the *in vivo* chick chorio-allantoic membrane (CAM) assay (Lokman et al., 2012) and the *in vivo* mouse/rodent graft model (Eklund et al., 2013). These two models are based on grafting tumor cells on the membrane of a growing chicken or in a specific site in mouse. If the chick model follows to some extent better the 3R rule (replacement, reduction, and refinement; Aske and Waugh, 2017) as considered as less sentient living beings due to not fully active nervous system, it is nevertheless necessary to develop alternative models that are closer to the human situation.

During the last decade, tissue engineering led to the development of artificial vessels which can be used for tissue vascularization in a 3D environment. These *in vitro* models may represent a promising alternative to animal models.

Regarding studies related to tumor–vessel interactions, it is important to note that cancer cells are involved in two phenomena: inducing vessel sprouting (angiogenesis) and transmigrating through the blood vessel wall (endothelial cells, smooth muscle cells, and matrix) for dissemination. The latter phenomenon is difficult to study in both CAM and mouse models. It is therefore necessary to develop *in vitro* models that are closer to the human situation. During the last decade, tissue engineering led to the development of artificial human vessels which can be used for tissue vascularization in a 3D environment. These *in vitro* models, which harbor all the histological components of a blood vessel (lumen, endothelium, smooth muscle cells, and matrix), must also retain its mechanical properties such as liquid-tightness, perfusability, and contractility.

### Tissue-Engineered Blood Vessels

Since the 1950s, synthetic tubes were the first choice for vascular reconstruction and grafts in patients with cardiovascular diseases. These conducts were made of polymer materials like expanded polytetrafluoroethylene (ePTFE), polyethylene terephthalate (Dacron), and polyurethane (Kannan et al., 2005), but all the functional characteristics of a blood vessel were not maintained in these tubes: they were not contractile and are not immune to thrombosis or inflammation. Motivated by these limitations, the development of 3D tissue-engineered blood vessels (TEBVs) has progressed significantly over the past two decades. Indeed, TEBVs tend to better match the biomechanical properties and the physiological responses of healthy blood vessels.

**FIGURE 2:**
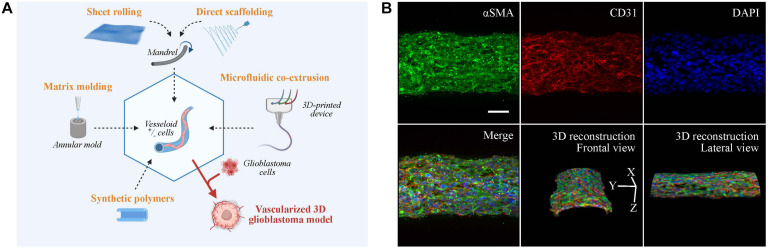
Vascular models in neuro-oncology research. **(A)** Fabrication strategies for engineering artificial vessels. Vascular models are synthesized by rolling, molding, scaffolding, or microfluidic co-extrusion using biomaterials with/without cells. Addition of glioblastoma cells creates a vascularized tumor model. **(B)** Confocal imaging of vesseloid (scale bar: 50 μm). Nuclei in blue (DAPI), αSMA in green, and endothelial marker in red (CD31). On top and merge panels, images correspond to a maximal intensity projection along the *z*-axis. Other panels are 3D-image reconstructions.

Tissue-engineered blood vessels can be not only used for vascular grafts but also for mechanistical studies related to the tumor–vessel interaction. For all the aforementioned reasons, engineering of artificial blood vessels is not an easy task and has been for a long time restricted to big diameter arteries (>6 mm) (Niu et al., 2014), and were usually made of synthetic polymers without endothelial of smooth muscle cell. Recently, efforts have been made to produce smaller blood vessels (<2 mm) by the use of various approaches (Song et al., 2018) (Figure 2A).

The production of tubular structures is achieved by three commonly used techniques: sheet rolling, tubular molding, and direct scaffolding. The sheet rolling technique is based on the creation of a sheet with the biomaterial of your choice and on the rolling of several sheets together for generating a tubular structure (Peck et al., 2011). Weinberg and Bell (1986) first described the use of a tubular mold that was filled with the desired cells and matrix. Finally, most of the biopolymers can be directly injected in a tubular form by direct scaffolding, but the major issue for all these techniques is the long-term culture of blood vessel. As an example, two pioneered groups (Roger Kamn and Lance Munn) developed in 2011 and 2012 microfluidic channels surrounded by 3D hydrogels using microfabricated silicone molds (Song and Munn, 2011; Shin et al., 2012). The point was to put endothelial cells in 2D culture on top of these hydrogels to mimic vessel sprouting and invasion inside a tissue. These models were subsequently improved by mixing stroma and tumor cells inside the hydrogel for tumor angiogenesis studies. A recent paper by Andrique et al. (2019) used a co-extrusion microfluidic device to produce small-diameter artificial vessels (<500 μm) with both endothelial and smooth muscle cells, surrounded by a scaffold made of biocompatible alginate polymer. These “vesseloids” are rapidly formed (only 1 day of culture) easy to handle, perfusable, liquid-tight, and retain their vascular functions (contractility, response to inflammatory stimuli) (Figure 2B). Vesseloids may be used as trunks for angiogenic sprouts to emerge which are useful for organ or tumor vascularization.

### Controlled Microenvironment in 3D *in vitro* Models

3D co-culture systems including tumors cells, endothelial cells (blood vessels), and other micro-environmental components have emerged for reproducing tumor–stroma interactions. It was shown that tissue stiffness modulate tumor growth and nutrient transport (Massa et al., 2017). This is critical and must be considered for drug testing in cancer therapy. The major advantage of 3D co-culture models relies on the fact that various parameters can be controlled: type and density of the ECM components (e.g., Matrigel, collagen, PEG, fibronectin, and gelatin methacryloyl), stiffness, hypoxia and gas exchange, and the inclusion of various cell types such as macrophages, astrocytes, or vascular cells.

3D systems, which do not include the vascular component, already improve phenotypic properties, gene expression, and drug response. The importance of the environment in drug delivery testing was already highlighted by Seo et al. (2014). They explained that new anti-cancer drugs are usually tested in 2D tissue culture which neglects the complexity of the 3D micro-environment (Langhans, 2018). The development of 3D *in vitro* tissue-engineered models will help to refine the drug response and contribute to the improvement of anti-cancer therapies. More recently, striking differences in gene expression between 2D and 3D culture of GBM have been reported (Ma et al., 2018) which mirrored the phenotypic differences. This is in agreement with another study (Chaicharoenaudomrung et al., 2020) where GBM cells were cultured in Ca-alginate 3D scaffolds before next-generation sequencing (Illumina), and this uncovered cellular pathways (Map kinase, autophagy, and cell metabolism) for 3D different to 2D cultures. Musah-Eroje and Watson (2019) developed a new 3D model of GBM which seems to more accurately reflect the complexity of the GBM micro-environment. Compared with regular 2D cultures or spheroids, they showed that GBM cells in the3D model were more resistant to temozolomide and that this resistance was potentiated by hypoxia. One unresolved issue regarding 3D brain organoid models is the lack of functional vascularization. Recently, Cakir et al. (2019) developed with success *in vitro* functional vasculature-like networks in human cortical organoids (hCOs) from human embryonic stem cells (hESCs). In this work, endothelial reprogramming in hCOs induced the formation of organoids with vascular-like structures. These vascular structures are functional and exhibit BBB characteristics. Close to this work, a recent model of vascularized human cortical organoids (vOrganoids) was developed by Shi et al. (2020) (Vascularized human cortical organoids (vOrganoids) model cortical development *in vivo* 2020 PLOS One). These vOrganoids (human cortical cell types with vasculature structure) presented bidirectional electrical transmission trough functional synapses, and their transplantation in the mouse cortex resulted in the survival of the graft. All these innovative 3D models represent useful models for studies related to physiology or pathology and may be useful as a model for therapeutic studies.

### 3D Co-Culture Models of Tumor and Endothelial Cells

The ultimate step of these 3D tumor models is the inclusion of endothelial and/or pericytes/smooth muscle cells to mimic blood vessels to produce vascularized tissues. A number of these works were done in tumor types other than glioblastoma. For example, Silvestri et al. (2020) developed a tissue-engineered model of a 3D co-culture of microvessels and mammary tumor organoids. They first fabricated a collagen gel scaffold with cylindrical channels filled with endothelial cells as a microvessel device. After perfusion and verification of the microvessels permeability, mammary tumor organoids were introduced inside of the 3D collagen scaffold and tumor cell–endothelial cell interactions were analyzed by live imaging. Others went further and developed a tri-culture metastatic model of breast cancer (Cui et al., 2020). By stereolithography 3D printing, a complex tripartite tissue was created which is composed of bone, vessels, and breast cancer cells. This model was used to study mechanisms of bone metastasis. Wang et al. (2014) have developed a 3D model to observe tumor invasion with high spatial and temporal resolution. Furthermore, intravasation and extravasation were observed at high spatial resolution using microvessels embedded in collagen 3D matrix and mixed with cancer cells (Langhans, 2018). As for glioblastoma, McCoy et al. (2019) generated GBM spheroids of uniform size distribution, and embedded them into collagen hydrogels to investigate GBM invasion into the ECM of the perivascular niche. They also showed by co-culturing endothelial and GBM cells that GBM cells have a high stemness potential and invasion capacity dependent on IL-8 signaling. Thus, tumor vasculature models may be very useful to shed light into the complex interactions between the vasculature and tumor cells.

## Revival of Anti-Angiogenic Therapy: Combination With Other Drugs

### Anti-Angiogenic Background

To date, bevacizumab is the only anti-angiogenic drug admitted for GBM management. Based on the results of three different clinical studies (Table 1), bevacizumab was approved by the Food and Drug Administration (FDA), the Japanese Ministry of Health, Labour and Welfare (MHLW), and other countries as a combination treatment with standard therapy and as a single agent for relapsed or progressive GBM after previous therapy. However, the OS was not prolongated in these studies; bevacizumab showed nevertheless some benefit in decreasing the use of corticosteroids, the adverse effects of which impair patient’s quality of life. However, the European Medicine Agency (EMA) has not approved its indication, estimating that the benefit–risk assessment is not in favor of its prescription in the management of GBM. This is also supported by the National Institute for Health and Care Excellence (NICE) guidelines (NICE, 2018). It is important to emphasize that bevacizumab cannot be used a month before and after brain surgery. This must be considered when using this treatment. Of note, the pharmacokinetics and bioavailability of bevacizumab is limited since it cannot cross the intact BBB which is nevertheless partially disrupted to allow drug penetration to some extent.

**TABLE 1:** Pivotal clinical trials supporting the approval indication.

Study name or ID	Design	Treatment arms	Median PFS (1) vs (2)	Median OS (1) vs (2)
AVAglio (NCT00943826) (Chinot et al., 2014) (new dig GBM)	Randomized, double-blinded, placebo-controlled Phase III (N = 921)	(1) RT + TMZ + Placebo (*N* = 463) (2) RT + TMZ + Bev (*N* = 458)	*6.2 (6.0–7.5) vs. 10.6 (10.0–11.4) months; HR = 0.64 (0.55–0.74); *p* < 0.001	*16.7 (15.4–18.4) vs. 16.8 (15.5–18.5) months; HR = 0.88 (0.76–1.02); *p* = 0.1
RTOG0825 (NCT00884741) (new dig GBM)	Randomized, double-blinded, placebo-controlled Phase III (*N* = 637)	(1) RT + TMZ + Placebo (*N* = 317) (2) RT + TMZ + Bev (*N* = 320)	*7.3 (5.6–7.9) vs. 10.7 (10.0–12.2) months; HR = 0.79 (0.66–0.94); *p* = 0.004	*16.1 (14.8–18.7) vs. 15.7 14.2–16.8) months; HR = 1.13 (0.93–1.37); *p* = 0.11
EORTC26101 (NCT01290939) (Wick et al., 2017) (recurrent GBM)	Randomized Phase III (*N* = 437)	(1) Lomustine alone (*N* = 149) (2) Lomustine + Bev (*N* = 288)	^#^1.5 (1.5–2.5) vs. 4.2 (3.7–4.3) months; HR = 0.49 (0.39–0.61); *p* < 0.001	*8.6 (7.6–10.4) vs. 9.1 (8.1–10.1) months; HR = 0.95 (0.74–1.21); *p* = 0.65

*RT, radiotherapy; TMZ, temozolomide; Bev, bevacizumab; PFS, progression-free survival; OS, overall survival. ^∗^Primary outcome measure. ^#^Secondary outcome measure.*

### Revival of Bevacizumab and Anti-Angiogenic Therapy

Bevacizumab alone is certainly not a curative treatment for GBM and this raises ethical issues related to the benefit–risk of this treatment between the improvement of the patients’ quality of life and frequent occurrence of serious side effects. Security data of clinical trials, as pharmacovigilance studies, have shown frequent and serious cardiovascular effects (hemorrhages, thromboembolic events, and heart failure) and hematological disorders (neutropenia, leukopenia, and thrombocytopenia) (Chinot et al., 2014; Gilbert et al., 2014; Wick et al., 2017). On theoretical grounds, the use of anti-angiogenic drugs may be justified due to physiopathological consideration (high expression of VEGF, BBB dysfunction, edema leading to hemorrhages, cognitive impairment, tumor growth, and cell invasion). Better outcomes may be observed when anti-angiogenic therapy is combined with inhibitors of tumor cell invasion or in combination with immunotherapy as documented in some studies (Kang et al., 2015; Piao et al., 2016; Gravina et al., 2017; Daubon et al., 2019).

A short review of the last-5-years published clinical trials is shown in Table 2, using the following parameters from PubMed: key words = Glioblastoma AND Antiangiogenic; filters = Abstract available, Clinical Trial; date = between 2015 and 2020. As no clinical trials of phase III were found, articles related to phase II studies, describing new associations or new indications with bevacizumab or new individual anti-angiogenic drugs, were selected if median progression-free survival (mPFS) or the median overall survival (mOS) outcomes were available and if the trial was referenced in https://clinicaltrials.gov/. mPFS and mOS reflect more robust outcomes than response rate. Eighteen clinical trials were found using these inclusion criteria.

**TABLE 2:** Phase II clinical trials recently published.

Study name or ID	Indication	Design	Treatment arms	Outcomes (months)	
				mPFS	mOS
NCT01349660 (Hainsworth et al., 2019)	Relapsed or refractory GBM following first-line therapy	Non-randomized, single group assignment, open label (*N* = 76)	BKM 120 (buparlisib) *per os* + Bev (1) Prior anti-angiogenic therapy (*N* = 19) (2) Without previous anti-angiogenic therapy (*N* = 57)	*(1) 2.8 (1.6–5.3) *(2) 5.3 (3.6–9.2)	^#^*(1)* 6.6 (4.0–14.6) ^#^*(2)* 10.8 (9.2–13.5)
NCT01753713 (Sharma et al., 2019)	Recurrent or progressive GBM following first-line therapy	Non-randomized, parallel assignment, open label (*N* = 33)	(1) Dovitinib *per os* in anti-angiogenic naïve patients (*N* = 19) (2) Dovitinib *per os* in progressed GBM on previous anti-angiogenic therapy (*N* = 14)	^#^*(1)* 2.0 (1.3–3.7) ^#^*(2)* 1.8 (0.9–1.8)	^#^*(1)* 8.0 (4.4–11.7) ^#^*(2)* 4.3 (2.6–6.7)
NCT00892177	Recurrent or progressive GBM	Randomized, parallel assignment, double blinded (*N* = 12(1)	*(1)* Bev + dasatinib *per os* (*N* = 83) *(2)* Bev + placebo (*N* = 38)		^#^*(1)* 7.3 (6.2–9.7) vs. *(2)* 7.9 (6.6–11.3); HR = 0.92 (0.61–1.4); *p* = 0.7
REGOMA (NCT02926222) (Lombardi et al., 2019)	Relapsed GBM after surgery	Randomized, parallel assignment, open label (*N* = 119)	*(1)* Regorafenib *per os* (*N* = 59) *(2)* Lomustine *per os* (*N* = 60)		*(1) 7.4 (5.8–12.0) vs *(2)* 5.6 (4.7–7.3); HR = 0.5 (0.33–0.75); *p* = 0.0009
ARTE (NCT01443676) (Wirsching et al., 2018)	Newly diagnosed GBM in elderly patients	Randomized. parallel assignment, open label (*N* = 75)	*(1)* RT (*N* = 25) *(2)* RT + Bev (*N* = 50)	^#^*(1)* 4.8 vs *(2)* 7.6; HR = 0.36 (0.20–0.65); *p* = 0.001	*(1) 12.1 vs *(2)* 12.2; HR = 1.09 (0.63–1.89); *p* = 0.75
ATAG (NCT02898012)(Reyes-Botero et al., 2018)	GBM in elderly patients with a Karnofsky performance status < 70	Non-randomized, single group assignment, open label (*N* = 66)	TMZ + Bev (*N* = 66)	^#^3.8 (3.2–4.8)	*6 (4.8–6.9)
HERBY (NCT01390948) (Grill et al., 2018)	Newly diagnosed GBM in pediatric and adolescent patients	Randomized, parallel assignment, open label (*N* = 12(1)	*(1)* RT + TMZ (*N* = 59) *(2)* RT + TMZ + Bev (*N* = 62)		^#^*(1)* 20.3 (14.8–33.8) vs. *(2)* 18.3 (16.2–25.7); HR = 1.23 (0.72–2.09); *p* = 0.46
NRG/RTOG (NCT01609790) (Reardon et al., 2018)	Recurrent GBM	Randomized, parallel assignment, double blinded (*N* = 115)	*(1)* Bev + placebo (*N* = 58) *(2)* Bev + trebananib (*N* = 57)	^#^*(1)* 4.8 (3.8–7.1) vs. *(2)* 4.2 (3.7–5.6); HR = 1.51 (1.02–2.24); *p* = 0.04	^#^*(1)* 11.5 (8.4–14.2) vs *(2)* 7.5 (6.8–10.1); HR = 1.46 (0.95–2.27); *p* = 0.09
NCT01738646 (Ghiaseddin et al., 2018)	Recurrent GBM	Non-randomized, single group assignment, open label (*N* = 40)	Bev + vorinostat (*N* = 40)	^#^3.7 (2.9–4.8)	^#^10.4 (7.6–12.8)
NCT00704288 (Cloughesy et al., 2018)	Recurrent or progressive GBM following previous anti-angiogenic therapy	Non-randomized, single group assignment, open label (*N* = 222)	Cabozantinib *per os* (*N* = 70) *(1)* 140 mg/day (*N* = 12) *(2)* 100 mg/day (*N* = 58)	^#^2.3 overall (1) 3.3– 2) 2.3	^#^4.6 (3.0–5.6) overall (1) 4.1– 2) 4.6
NCT00704288 (Wen et al., 2018)	Recurrent or refractory GBM following non–anti-angiogenic therapy	Non-randomized, single group assignment, open label (*N* = 222)	Cabozantinib *per os* (*N* = 152) *(1)* 140 mg/day (*N* = 34) *(2)* 100 mg/day (*N* = 118)	^#^3.7 overall	^#^(1) 7.7 ^#^2) 10.4
GO27819 (NCT01632228) (Cloughesy et al., 2017)	Recurrent GBM	Randomized, parallel assignment, double blinded (*N* = 129)	*(1)* Bev + onartuzumab (*N* = 64) *(2)* Bev + placebo (*N* = 65)	*(1) 3.9 vs. *(2)* 2.9; HR = 1.06 (0.72–1.56); *p* = 0.74	^#^*(1)* 8.8 vs. *(2)* 12.6; HR = 1.45 (0.88–2.37); *p* = 0.14
NCT01846871 (Kalpathy-Cramer et al., 2017)	Recurrent GBM	Non-randomized, single group assignment, open label (*N* = 10)	Tivozanib	^#^2.3 (1.5–4)	^#^8.1 (5.2–12.5)
NCT01067469 (Weathers et al., 2016)	Recurrent GBM	Randomized, single group assignment, open label (*N* = 69)	*(1)* Bev (*N* = 36) *(2)* Bev low doses + lomustine (*N* = 33)	*(1) 4.11 (2.96–5.55) *(2) 4.34 (2.96–8.34)	^#^*(1)* 8.3 (6.42–11.58) ^#^*(2)* 9.6 (6.26–16.73)
GLARIUS (NCT00967330) (Herrlinger et al., 2016)	Newly diagnosed GBM and a non-methylated MGMT promoter	Randomized, parallel assignment, open label (*N* = 182)	*(1)* Bev + irinotecan (*N* = 122) *(2)* TMZ (*N* = 60)	^#^*(1)* 9.7 (8.7–10.8) vs. *(2)* 6.0 (2.7–6.2); HR = 0.59 (0.42–0.82); *p* = 0.001	^#^*(1)* 16.6 (15.4–18.4) vs. *(2)* 17.3 (14.8–20.4); HR = 0.96 (0.68–1.35); *p* = 0.83
NCT00667394	Recurrent GBM	Non-randomized, parallel assignment, open label (N = 41)	Bev + tandutinib	^¤^4.1	^¤^11
NCT00720356 (Raizer et al., 2016)	Newly diagnosed GBM and a non-methylated MGMT promoter	Non-randomized, single-group assignment, open label (*N* = 46)	Bev + erlotinib	^¤^9.2 (6.4–11.3)	*13.2 (10.8–19.6)
NCT00859222 (Lee et al., 2015)	Recurrent GBM	Non-randomized, single group assignment, open label (*N* = 24)	Bev + panobinostat	^#^5 (3–9)	^#^9 (6–19)

mPFS, median progression-free survival; mOS, median overall survival; Bev, bevacizumab; RT, radiotherapy; TMZ, temozolomide.* Primary outcome measure. ^#^Secondary outcome measure. ^¤^Post hoc analysis.

### New Investigations for Bevacizumab

Certain populations are under-represented in global clinical trials. This has led to the investigation of the efficacy of bevacizumab in newly diagnosed GBM (nGBM) in the elderly (ARTE, Wirsching et al., 2018 and ATAG, Reyes-Botero et al., 2018 studies) and in pediatric populations (HERBY study, Grill et al., 2018). The ARTE study showed that when radiotherapy associated with bevacizumab, it did not prolong mOS compared with radiotherapy only (12.2 vs 12.1 months; HR = 1.09; *p* = 0.75). However, mPFS was favorable with bevacizumab in restricted *per* protocol analyses (7.6 vs 4.8 months; HR = 0.36; *p* = 0.001) (Wirsching et al., 2018). In combination with temozolomide, bevacizumab seemed active in the ATAG study and had an acceptable adverse effect profile (Reyes-Botero et al., 2018). As for the pediatric population, adjunction of bevacizumab to the current therapy did not improve mOS (18.3 vs 20.3 months; HR = 1.23; *p* = 0.46) (Grill et al., 2018). The results in the pediatric population differ from adults, and, thus, further research is needed.

### New Associations With Bevacizumab

Glioblastomas are associated with increased stimulation of different signaling pathways. Trials have been run with bevacizumab combined with molecules interfering with these pathways. The association of bevacizumab with BKM 120–buparlisib, an oral PI3K inhibitor, did not improve outcome (mPFS: 2.8 to 5.3 months; mOS: 6.6 to 10.8 months) and increased the adverse drug effect profile (Hainsworth et al., 2019). In another study, mOS in the arm of bevacizumab combined with dasatinib, a Src signaling inhibitor, is similar to the placebo arm (7.3 vs 7.9 months; HR = 0.92; *p* = 0.7) (Galanis et al., 2019). In two trials, addition with bevacizumab to the histone deacetylase inhibitors vorinostat (Ghiaseddin et al., 2018) and panobinostat (Lee et al., 2015) failed to improve outcome compared with control (mPFS: 3.7 and 5 months; mOS: 10.4 and 9 months, respectively). When bevacizumab is combined with onartuzumab, a monovalent MET inhibitor, it did also not improve patient outcome versus bevacizumab plus placebo alone (mPFS: 3.9 vs 2.9 months; HR = 1.06; *p* = 0.74 and mOS: 8.8 vs 12.6 months; HR = 1.45; *p* = 0.14) (Cloughesy et al., 2017). The GLARIUS trial aimed to study the association of bevacizumab with irinotecan, a topoisomerase 1 inhibitor, comparing with temozolomide alone in nGBM with un-methylated MGMT promoter. This resulted in a superior mPFS with the drug combination (9.7 vs 6.0 months; HR = 0.59; *p* = 0.001) without improving mOS (16.6 vs 17.3 months; HR = 0.96; *p* = 0.83) (Herrlinger et al., 2016).

Another strategy aims at an additive effect by targeting both the vasculature and tumor cells and by combining bevacizumab with inhibitors of other growth factor pathways. Trebananib is a Fc fusion protein that targets Ang1 and Ang2; however, its association with bevacizumab failed to improve outcome when compared with bevacizumab plus placebo alone (mPFS: 4.2 vs 4.8 months, HR = 1.51, *p* = 0.04; and mOS 7.5 vs 11.5 months, HR = 1.46, *p* = 0.09) (Reardon et al., 2018). The association between bevacizumab and tandutinib, an oral FLT3, c-Kit, and PGDFRβ inhibitor, showed some benefit (*post hoc* mPFS: 4.1 months; *post hoc* mOS: 11 months) but showed a high toxicity (Odia et al., 2016). One trial with patients presenting a nGBM with an unmethylated MGMT promoter investigated the combination with erlotinib, an EGFR tyrosine kinase inhibitor. This association did not also increase survival (mOS: 13.2 months – estimated mOS should have reached 17.9 months to show an increase in survival) (Raizer et al., 2016). This indicates that, during these last 5 years, no new drug combination with bevacizumab showed substantial clinical benefit and even increased toxicity. Furthermore, when comparing the use of low doses of bevacizumab to standard doses in patients with rGBM, it did not improve survival (mPFS: 4.34 vs 4.11 months; mOS: 9.6 vs 8.3 months) (Weathers et al., 2016).

Combination with immunotherapy also did not provide significant benefit. Recently nivolumab was investigated in a phase III randomized clinical trial with or without bevacizumab in patients with rGBM; nivolumab arm did not improve mOS [9.8 (8.2–11.8) vs 10.0 (9.0–11.8) months; HR = 1.04 (0.83–1.30), *p* = 0.76] and showed a lower mPFS [1.5 (1.5–1.6) vs 3.5 (2.9–4.6) months; HR = 1.97 (1.57–2.48), *p* > 0.001] (Reardon et al., 2020).

### New Anti-Angiogenic Drugs

Bevacizumab is the first representative of a drug family that interfered with the VEGF pathway. Others are represented by VEGF receptor tyrosine kinase inhibitors. A great advantage of these drugs is the oral administration which increases the patient’s observance. In 2013, a phase III clinical trial studied the efficacy of cediranib, VEGFR, PDGFR, and c-Kit inhibitor, in combination with lomustine versus lomustine alone in patients with rGBM, but results did not show improvement of PFS (Batchelor et al., 2013). In a trial that compared dovitinib, a FGFR and VEGFR inhibitor, as second-line treatment after prior anti-angiogenic therapy by bevacizumab, no efficacy in prolonging mPFS (1.8 vs 2 months) was seen (Sharma et al., 2019). Tivozanib also showed limited activity in rGBM (mPFS: 2.3 months; mOS: 8.1 months), but the patient number in the trial (*N* = 10) was low (Kalpathy-Cramer et al., 2017). Two studies on cabozantinib, which inhibits MET and VEGFR2, on a global population did show some positive outcome in the rGBM group not treated previously with anti-angiogenic drugs (mPFS: 2.3 and 3.7 months; mOS: 4.6 and 10.4 months) (Cloughesy et al., 2018; Wen et al., 2018). Furthermore, the REGOMA study showed encouraging therapeutic benefit with regorafenib compared with lomustine alone (mOS: 7.4 vs 5.6 months; HR = 0.5; *p* < 0.001) (Lombardi et al., 2019).

### Drug Delivery

Therapy development for GBM is challenging. This is due to resistances to radio- and chemotherapy because of the presence of glioblastoma stem-like cells (Safa et al., 2015). Furthermore, the CNS is composed of natural barriers which impair drug delivery into the brain. As such, the BBB allows the passive transport of gas and liposoluble molecules. BBB’s tight junctions regulate, furthermore, paracellular transport.

Current pharmacological treatments for GBM are administered systemically by intravenous injection or orally. Oral route simplifies patient treatment by proposing several pharmaceutical options. Parenteral route allows a short action period and a controlled dosage. However, tissue diffusion into the brain is hampered and toxic side effects occur because of their systemic action and the possibility to reach the brain tissue only when the BBB is altered. To overcome these problems, permeability of drugs can be enhanced by increasing liposolubility or integrating them into liposomes or nanocarriers. On the other hand, BBB can be temporarily disrupted by therapeutic ultrasound whereas a hyperosmotic disruption did not improve the drug penetration (Kobrinsky et al., 1999; Idbaih et al., 2019).

Another possibility is to administer topically medicinal products using injectable or implantable devices with sustained drug release. Local delivery strategies aim at increasing the concentration of the drug at the tumor site, at decreasing alterations related to enzymatic metabolization, and at reducing the systemic side effects. After surgery, the resection cavity represents an accessible implantation site near non-surgically resectable cells. The only approved strategy by the FDA for nGBM and rGBM is the carmustine-impregnated biodegradable Gliadel wafer. However, these implants did not improve outcomes and presented higher local toxicity (brain edema, seizures) (De Bonis et al., 2012). Hydrogels are a 3D matrix composed of a hydrophilic polymer network. Injectable hydrogel is a reservoir-system similar to soft tissue that can contain a large panel of drugs able to diffuse into the surrounding tissue (Basso et al., 2018). Antineoplastic drugs can be administered directly into the cavity or in the cerebrospinal fluid via an intrathecal injection device (Ommaya reservoir) for therapeutic delivery (Ommaya, 1984). However, the drug concentration decreases as the diffusion distance increases, and thus, this approach is of limited use in highly infiltrating tumors. Moreover, long-term use of these medical devices may cause complications including infections and hemorrhages. Convection-enhanced delivery has been developed to increase local delivery by enhancing diffusion by a bulk flow to maintain a pressure gradient (Bobo et al., 1994). Despite an acceptable safety profile, this method did not improve clinical outcomes of patient with GBM (Oberoi et al., 2016). An alternative method is the use of the intranasal delivery, which is non-invasive because of the anatomical proximity of these structures. Intranasal administration of a telomerase inhibitor in a rat model extended animal survival (Hashizume et al., 2008).

### Perspectives for Anti-Angiogenic Drugs

There are new avenues to be explored for anti-vascular therapy. They can be used to enhance the activity of other therapies. For example, local hypoxia induced by bevacizumab could activate evofosfamide, a hypoxia-activated alkylating prodrug (Duan et al., 2008). Evofosmamide was studied on a phase I clinical trial for the treatment of rGBM following previous bevacizumab therapy, and results appeared to be favorable for being studied in a phase II trial (Brenner et al., 2018). Furthermore, anti-angiogenic therapy could be of more benefit in some GBM subgroups. In a retrospective study of the AVAglio Trial, it has been shown that bevacizumab treatment led to a prolongation of OS of 4.3 months compared with placebo (17.1 vs 12.8 months; multivariable HR = 0.42; *p* = 0.001) in patients with proneural and IDH-1 wild-type nGBM (Sandmann et al., 2015). To date, the use of anti-angiogenic drugs should preferably be part of personalized care for patients.

## General Conclusion

The vasculature plays an important role in the brain in normal and pathological conditions. In this article, we reviewed some recent literature on this subject. In a healthy tissue, endothelial cells are considered gatekeepers in all vessel types, for controlling diffusion of soluble factors or immune cells, by using para- or transcellular pathways. In GBM, however, vessels present maturation defect and chronic hyper-permeability, leading to vessel leakage, and poor vessel perfusion and delivery of nutrients. Pericytes and astrocytes have a central role in controlling physiology of normal and GBM NVUs. Pericytes, which are positioned along capillaries, help GBM cells to invade distant sites along blood vessels, as observed for reactive astrocytes. Importantly, GBM cells displace the astrocytic endfeet during co-option, disrupting endothelial cell junctions, and participating in blood leakage and hemorrhage.

The tumor–vessel interaction can also be modeled using *in vitro* bioengineered blood vessels. For now, no perfect 3D co-culture model exists. However, recent efforts have been made at developing 3D artificial vessels and 3D co-culture models using co-cultures of cancer cells and artificial blood vessels. Regarding vascularized 3D GBM models, researchers departed from 2D co-cultures to spheroids and are now able to reproduce small brain organoids with or without a functional vascular network. The main challenge for brain organoids is the co-culture of multiple cell types including neurons, astrocytes, oligodendrocytes, and microglia (Cakir et al., 2019). Another difficulty is the reconstruction of a tissue resembling to the human brain with microglia and six cortical layers (Heide et al., 2018) which exhibits the functional characteristics of the human brain such as neuronal networks and functional synapses (Shi et al., 2020). Moreover, the presence of a stabilized functional vascular network is also critical which requires the improvement of the current models for better recapitulating the physiopathology of these models.

3D co-culture models of blood vessels have been recently developed which may be used as vascularization trunks for tumor models. 3D models are much more relevant to study these interactions because they better recapitulate cell behavior and also better mirror *in vivo* gene expression and signaling. 3D co-culture models represent an attractive alternative to animal models and may be used in drug screening to identify better therapies.

Anti-angiogenic therapy in GBM did not meet the initial high expectations when tested in clinical trials. There was no real clinical benefit in newly diagnosed and recurrent GBM (maybe with exception of regorafenib). However, if clinical trials allow to obtain a global vision of the therapeutic effect, they do not consider patient subgroups. When considered, this may allow a more precise vision of the therapeutic response. Another drawback is variable study design and the criteria for determining progression. Radiologic response criteria such as Macdonald or RANO criteria may be misleading in monitoring clinical responses to anti-angiogenic therapy. Thus, this is still not the end of the road for anti-angiogenic therapy in GBM and more promising data from clinical trials are expected to come.

## Author Contributions

All authors listed have made a substantial, direct, and intellectual contribution to the work, and approved it for publication.

## Conflict of Interest

The authors declare that the research was conducted in the absence of any commercial or financial relationships that could be construed as a potential conflict of interest.
